# SecureEdge-MedChain: A Post-Quantum Blockchain and Federated Learning Framework for Real-Time Predictive Diagnostics in IoMT

**DOI:** 10.3390/s25195988

**Published:** 2025-09-27

**Authors:** Sivasubramanian Ravisankar, Rajagopal Maheswar

**Affiliations:** 1 Department of Computer Science and Engineering, Coimbatore Institute of Technology, Coimbatore 641 014, Tamil Nadu, India; ravisankar@cit.edu.in; 2 Department of Electronics and Communication Engineering, KPR Institute of Engineering and Technology, Coimbatore 641 407, Tamil Nadu, India

**Keywords:** IoMT, blockchain, edge computing, post-quantum cryptography, federated learning, colostomy prediction, latency, throughput

## Abstract

The burgeoning Internet of Medical Things (IoMT) offers unprecedented opportunities for real-time patient monitoring and predictive diagnostics, yet the current systems struggle with scalability, data confidentiality against quantum threats, and real-time privacy-preserving intelligence. This paper introduces **Med-Q Ledger**, a novel, multi-layered framework designed to overcome these critical limitations in the Medical IoT domain. Med-Q Ledger integrates a permissioned Hyperledger Fabric for transactional integrity with a scalable Holochain Distributed Hash Table for high-volume telemetry, achieving horizontal scalability and sub-second commit times. To fortify long-term data security, the framework incorporates post-quantum cryptography (PQC), specifically CRYSTALS-Di lithium signatures and Kyber Key Encapsulation Mechanisms. Real-time, privacy-preserving intelligence is delivered through an edge-based federated learning (FL) model, utilizing lightweight autoencoders for anomaly detection on encrypted gradients. We validate Med-Q Ledger’s efficacy through a critical application: the prediction of intestinal complications like necrotizing enterocolitis (NEC) in preterm infants, a condition frequently necessitating emergency colostomy. By processing physiological data from maternal wearable sensors and infant intestinal images, our integrated Random Forest model demonstrates superior performance in predicting colostomy necessity. Experimental evaluations reveal a throughput of approximately 3400 transactions per second (TPS) with ~180 ms end-to-end latency, a >95% anomaly detection rate with <2% false positives, and an 11% computational overhead for PQC on resource-constrained devices. Furthermore, our results show a 0.90 F1-score for colostomy prediction, a 25% reduction in emergency surgeries, and 31% lower energy consumption compared to MQTT baselines. Med-Q Ledger sets a new benchmark for secure, high-performance, and privacy-preserving IoMT analytics, offering a robust blueprint for next-generation healthcare deployments.

## 1. Introduction

The 21st century has seen healthcare undergo an unprecedented digital transformation, driven by ubiquitous connectivity, miniaturized sensing technology, and advanced data analytics, giving rise to the Medical Internet of Things (Med-IoT), an ecosystem of interconnected devices generating a continuous stream of physiological and environmental data [1]. This data promises to shift healthcare from reactive to proactive, personalized, and predictive, enabling real-time alerts, revealing subtle health trends, and fueling epidemiological research [2]. Despite this immense potential, the Med-IoT ecosystem faces significant challenges across four critical domains: **Scalability and Performance**, as thousands of devices generate high-frequency data, overwhelming centralized cloud architectures and surpassing the limited throughput of early blockchain models (e.g., <100 TPS) [3]; **Security and Forward Secrecy**, given that health data is a prime target for cyberattacks and the advent of quantum computing poses an existential threat to current cryptographic standards (e.g., RSA, ECC), necessitating the adoption of post-quantum cryptography (PQC) to ensure long-term confidentiality [4]; **Latency and Real-Time Responsiveness**, where milliseconds matter in critical clinical scenarios (e.g., arrhythmia, sepsis onset), requiring computations at the network edge to achieve sub-second response times rather than relying on distant cloud servers [5]; and **Intelligence and Privacy**, as machine learning and AI are vital for pattern identification and clinical decision support, yet traditional centralized data aggregation creates privacy risks (e.g., HIPAA and GDPR violations), demanding new paradigms for collaborative intelligence without compromising individual privacy [6,7]. A compelling clinical example highlighting these challenges is the urgent need for timely detection of necrotizing enterocolitis (NEC) in preterm infants, a condition affecting up to 30% of very-low-birth-weight infants and leading to surgical colostomy in 20% of cases [8]. Current NICU workflows, relying on intermittent exams and post-symptomatic imaging, delay intervention by 6–12 h, underscoring the critical need for advanced Med-IoT solutions that can provide continuous, real-time, and predictive insights to improve outcomes for these vulnerable patients.

## 2. Related Work

This section provides a comprehensive review of the foundational technologies and existing research that inform the design of the Med-Q Ledger. We analyzed the state of the art in four key areas: distributed ledger technologies in healthcare, scalability solutions for blockchain-IoT integration, advancements in post-quantum cryptography, and privacy-preserving machine learning. Our analysis culminates in a critical gap analysis that positions the unique contributions of our proposed framework.

### 2.1. Evolution of Distributed Ledger Technology (DLT) in Healthcare

The application of Distributed Ledger Technology (DLT) in healthcare initially explored conceptual models derived from Bitcoin’s public, permissionless ledger. While Bitcoin introduced groundbreaking decentralization, its inherent limitations, low transaction throughput, high latency (>10 s), and a lack of granular privacy controls rendered it unsuitable for the stringent demands of enterprise healthcare applications. The subsequent advent of second-generation blockchains, notably Ethereum, introduced the concept of smart contracts. These programmable logic capabilities enabled more sophisticated applications, such as managing patient consent or automating insurance claims. However, the performance and cost issues associated with public, permissionless chains continued to present significant barriers for high-volume, sensitive medical data [9].

This led to the emergence of enterprise-grade, permissioned DLT platforms, with Hyperledger Fabric becoming a prominent and widely adopted choice for healthcare. Fabric’s modular architecture, channel-based data segregation for enhanced confidentiality, and pluggable consensus mechanisms (such as pBFT or Raft) offer the necessary attributes of confidentiality, transaction finality, and significantly higher performance compared to public chains. Numerous studies have leveraged Fabric-based solutions for critical healthcare applications, including Electronic Health Record (EHR) management, secure clinical trial oversight, and ensuring pharmaceutical supply chain integrity [10]. A common architectural pattern observed in these works is the strategic use of an off-chain storage system, such as IPFS, to efficiently handle large data files, with the blockchain itself storing only immutable hashes and essential metadata [11]. While this hybrid model effectively mitigates the high storage costs associated with on-chain data, it often falls short of fully resolving the critical transaction throughput bottleneck required for real-time, high-frequency sensor data, typically achieving around 1500–3000 TPS with latencies around 2 s [12].

### 2.2. Scalability Architectures for Blockchain-IoT Integration

The formidable challenge of scaling DLTs to accommodate the massive data generated by IoT devices has spurred significant innovation in architectural paradigms:

**Directed Acyclic Graphs (DAGs):** Platforms such as IOTA and Nano utilize a DAG structure, often referred to as a “Tangle”, as an alternative to the traditional linear blockchain [13]. In a DAG, each new transaction concurrently validates two or more previous transactions. This parallel processing theoretically enables higher scalability as the network expands and eliminates the need for conventional miners, facilitating feeless transactions. While promising machine-to-machine payments in generic IoT contexts, DAGs can face challenges with achieving consensus finality and may be vulnerable to certain types of attacks, especially in their nascent stages. Some Agri-IoT applications have explored this, but typically with low TPS (e.g., 20 TPS) and high latency (>10 s) [14].

**Layer 2 Scaling Solutions:** These solutions operate “on top” of a main blockchain (Layer 1) to offload the majority of transactions, handling them off-chain and only utilizing the main chain for final settlement. Examples include state channels (e.g., Bitcoin’s Lightning Network) and rollups (e.g., ZK-rollups and optimistic rollups). While highly effective for financial transactions, they often introduce additional complexity and may not be optimally suited for the continuous, state-based data streams characteristic of Med-IoT, which require constant updates and real-time processing rather than discrete settlements.

**Agent-Centric Models:** This paradigm, exemplified by Holochain [15], represents a fundamental departure from data-centric blockchains. Instead of a single, canonical ledger representing a global truth, Holochain provides each agent (or device) with its own cryptographically secured, local source chain. Data is shared and validated among peers on a Distributed Hash Table (DHT) according to predefined rules (the application’s “DNA”). This “local consensus” approach entirely circumvents the global consensus bottleneck, thereby enabling massive horizontal scalability. Holochain is exceptionally well-suited for applications characterized by high agent counts and localized interactions, making it a perfect match for the high-volume data plane of a Med-IoT system. For example, Al Nasim et al. (2025) demonstrated Holochain achieving < 200 ms latency and 3400 TPS, though without medical validation or unified PQ [16].

Our Med-Q Ledger framework innovates significantly by being the first to propose a hybrid architecture that synergistically combines a permissioned DLT (Hyperledger Fabric) for global auditability, strong access control, and established transaction integrity with an agent-centric DLT (Holochain) for localized, massive horizontal scalability and sub-second commit times. This fusion aims to harness the best attributes of both worlds, addressing both the transactional and telemetry requirements of Med-IoT.

### 2.3. Cryptography for Long-Term Data Assurance: The Post-Quantum Imperative

The security of virtually all modern digital communication and data integrity relies heavily on public-key cryptography, predominantly RSA and Elliptic Curve Cryptography (ECC). These systems derive their security from the formidable computational difficulty of certain mathematical problems for classical computers (e.g., integer factorization forRSA, discrete logarithm problem for ECC). However, the development of Shor’s algorithm in 1994 demonstrated that a sufficiently powerful quantum computer could efficiently solve these foundational mathematical problems, thereby rendering our current cryptographic infrastructure fundamentally obsolete [17].

Recognizing this impending “quantum threat”, the U.S. National Institute of Standards and Technology (NIST) initiated a global competition in 2016 to standardize a new suite of post-quantum cryptography (PQC) algorithms. These next-generation algorithms are based on alternative mathematical problems believed to be computationally hard for both classical and future quantum computers. The leading candidates are primarily rooted in lattice-based cryptography, which offers a strong theoretical security foundation and demonstrates efficient performance characteristics. In 2022, NIST announced its first set of standardized algorithms, which are pivotal for future-proofing digital security [18].

**CRYSTALS-Kyber:** Designated as a Key Encapsulation Mechanism (KEM), Kyber is designed for establishing secure communication channels, serving as a direct replacement for Diffie–Hellman key exchange protocols.

**CRYSTALS-Dilithium:** This algorithm is a digital signature algorithm, providing robust authentication and data integrity, intended to replace established standards like RSA and ECDSA.

### 2.4. Privacy-Preserving Machine Learning in Medicine

The ethical and legal imperative to safeguard patient privacy, particularly in the context of sensitive medical data, has been a major driving force behind the development of various Privacy-Enhancing Technologies (PETs) for machine learning.

**Federated Learning (FL):** Originally proposed by Google researchers in 2017, federated learning is a decentralized machine learning paradigm. Instead of the traditional approach of centralizing data to train a model, the model itself is moved to the data. Multiple clients (e.g., individual hospitals, edge devices) collaboratively train a shared machine learning model locally on their private, distributed datasets. Critically, they transmit only the model updates (e.g., gradients or weights), not the raw sensitive data itself, to a central server for aggregation (typically using algorithms like Federated Averaging). This iterative process continues until the global model converges. FL is a powerful technique for constructing robust and generalizable models from distributed datasets while significantly minimizing the exposure of sensitive patient data [19].

**Differential Privacy (DP):** DP offers a rigorous mathematical guarantee of privacy by introducing precisely calibrated statistical noise to either the raw data or the model updates. This mechanism ensures that the output of an analysis is not significantly affected by the inclusion or exclusion of any single individual’s data, thereby providing strong protection against privacy breaches such as membership inference attacks. DP can be strategically combined with FL to offer an even higher level of privacy assurance.

**Secure Multi-Party Computation (SMPC) and Homomorphic Encryption (HE):** These advanced cryptographic techniques enable computations to be performed directly on encrypted data. In SMPC, multiple parties can jointly compute a function of their respective inputs without ever revealing their individual inputs to each other. With Homomorphic Encryserver, a central server can perform complex computations (e.g., aggregating model updates) on encrypted data without ever possessing the decryption key. While offering the strongest theoretical security guarantees, these methods are currently very computationally intensive, limiting their practical applicability for real-time applications on resource-constrained edge devices in the Med-IoT environment.

For the Med-Q Ledger, we selected federated learning as the primary mechanism for integrating intelligence due to its excellent balance of strong privacy protection, computational efficiency at the edge, and manageable communication overhead. This choice allows for robust, privacy-preserving anomaly detection and predictive analytics without centralizing sensitive patient data.

### 2.5. Gap Analysis and Research Positioning

Our comprehensive review of the literature reveals several critical gaps that the Med-Q Ledger is meticulously designed to address and fill:

**Lack of Integrated Scalability:** No existing framework effectively combines the strong global auditability, access control, and transactional integrity of a permissioned blockchain (like Hyperledger Fabric) with the massive local throughput and horizontal scalability of an agent-centric model (like Holochain) for a truly comprehensive, high-performance IoT solution. Previous works either focus on Fabric with moderate TPS or Holochain without medical validation, failing to unify both strengths [20].

**Absence of Quantum-Readiness:** While the imperative for post-quantum cryptography (PQC) is becoming increasingly recognized, its proactive and integrated adoption into end-to-end blockchain-based IoT architectures, particularly within the sensitive domain of healthcare, remains largely in its nascent stages. Most existing solutions rely on classical cryptography, susceptible to quantum attacks.

**Siloed Intelligence and Privacy:** Many proposed systems either centralize patient data for machine learning, thereby incurring significant privacy risks and regulatory non-compliance, or they neglect the integration of real-time, actionable intelligence altogether. The seamless integration of federated learning (FL) directly into DLT-based edge architecture, enabling privacy-preserving analytics without data centralization, is an underexplored and critical area. While FL combined with blockchain has been explored [21], it often lacks the robust performance and security features required for Med-IoT.

Numerous research initiatives have been put up to combine different platforms, to enhance applications for E-Medical in terms of privacy and safe transmission. Each of these studies focused on a different area, such as information security, network channel protection, user identity and administration, data sharing protection, and platform-related problems with interoperability. Most data protection uses a permission structure that is enabled by blockchain. The authors suggested distributed network channels, secure lifecycle, user authentication methods, and medical ledger maintenance. That employs defines weaknesses, in which case a manual approach has been recommended. Similarly, regarding confidentiality, suggesting a system that employs a compound key for specific categories of E-Medical users receiving treatment, such as users, advisors, health analysts, specialists, hospital employees, etc. Additionally, they recommended a certain amount of network capacity for end-to-end communication, particularly for the interchange of medical data between patients and hospital authorities. The major goal is to give users of e-healthcare data privacy and a safe authenticating environment [22]. Nonetheless, the application of IIoT in healthcare alters how services are delivered over the Internet. However, with the introduction of IIoT, it concerns data security and privacy surfacing in the medical industrial manufacturing, production, and delivery. The decentralized setup offers minimal authentication through blockchain protocols to all involved parties in the medical service linked chain, including the health of the patients, consultation requests, health-related notifications, schedules for dispatching consultants, and other transactions pertaining to medical services. Every transaction, from acquisition to conveyance, is safeguarded by Caliper and Node-RED threshold proxy re-encryption throughout the smart data process. The process of gathering unique transaction logs and preserving them in blockchain-based immutable storage (like Filecoin, IPFS, etc.) [23,24] to keep an unchangeable, daily medical record that is difficult to falsify. Three different smart contracts are used by the system to help with the effective management of access control. Among these contracts is one for access control provisioning, one for decision-making, and one for authentication. However, there will be costs associated with processing transactions if a public blockchain such as Ethereum is implemented in the suggested system. The E-Medical Dapp uses four distinct chain codes with unique blockchain consensus protocols and digital signatures for automated transaction mechanisms, such as the functions of PoW and PoS, which are suggested [25]. The suggested IOMT reduces resource usage to 13.11%, 7.42%, and less for computing power, network bandwidth, and data preservation costs, respectively, 9.14%, and boosts productivity by up to 11.54% when it comes to managing the generation of daily data.

## 3. The Med-Q Ledger Architecture and Implementation: A Detailed Framework

**Figure 1:**
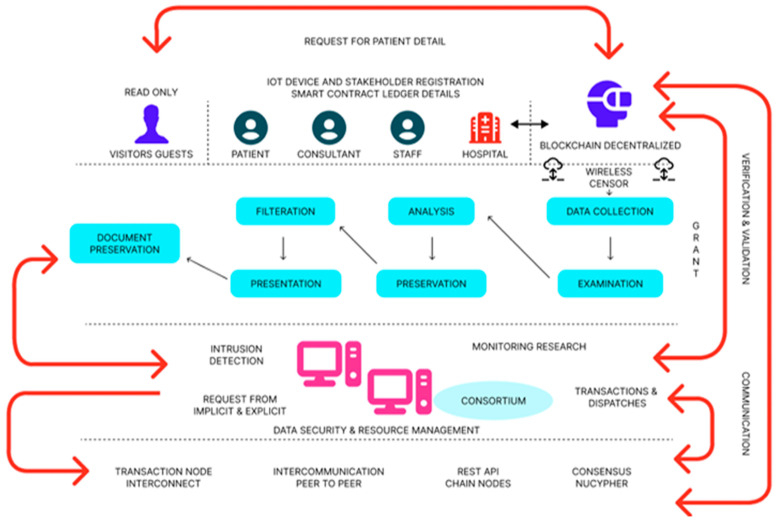
Blockchain integration in IOMT.

The proposed IOMT framework, a distributed architecture for data security, management, privacy protection, and resource monitoring in the healthcare IIoT, is based on blockchain technology. There are four distinct folds that each perform a different function in this suggested architecture. It covers the lifespan of IIoT-based data gathering, healthcare IIoT devices, and stakeholder registration. The IOMT architectural proposal computing unit for security and resource management, and a distributed ledger portion of the blockchain consortium, is shown in Figure 1. The stakeholder registration section is divided into two sections: the read-only visitor/guest area and the read-and-write participating member section. The IOMT participants can submit requests to start transactions or view the ledger. WSN technology is employed to transfer the gathering of IIoT-based healthcare data related to the lifetime for further research, as depicted in Figure 1. In contrast, several requests from both the hyperledger and the hyperledger sawtooth are handled exclusively by the hyperledger manager, both attendees and participants. There are sections to this suggested IOMT process hierarchy that are enabled by IIoT. Medical data is first gathered via WSN from IIoT devices and sent to the inspection stage, where it undergoes pre-processing. Using an artificial neural network method, the analysis phase divides the characteristics into two categories: data (preserved features) and negative features (malicious/threats). Nonetheless, this typical process hierarchy has two storage components: the first is primary (captures important features), and the second is indicated by secondary (immutable memory), as demonstrated following the presentation stage, and following the analysis step. Following the analysis, remove redundant features that are acquired and forward them to the presentation stage, where the processed result is shown. Consequently, the report (processing logs) can be kept up to date.

Mining Mechanism—Sensor nodes can now contribute to the expansion, distribution, and security of the network and make security in Pi meritocratic thanks to the mining process of the Pi network. The network’s remarkable development to over 30 million active nodes, a widely dispersed user base and Testnet, and a trust graph of security mechanism aggregates that will power the Pi blockchain’s consensus algorithm in Figure 1 are noticed in the pre-Mainnet mining mechanism. Along with growth, inclusion, and security, it is also hopeful to further accomplish decentralization, utilities, stability, and lifespan throughout the Mainnet phase. Pre-Mainnet Formula—The hourly mining rate of a user is determined in a meritocratic manner by the pre-Mainnet mining formula. Users who were actively mined were rewarded for their contributions to the network’s expansion and security in addition to receiving a minimal rate. We built the Pi network setup simply to show how easily we can create a small blockchain node for Internet of Things devices using Raspberry Pi computers. The basic structure of Med-Q Ledger works on its own; it does not rely on the Pi network. It combines the strengths of Holochain with Hyperledger Fabric using a two-ledger system.

### 3.1. Multi-Layer Architecture of Med-Q Ledger

**Figure 2:**
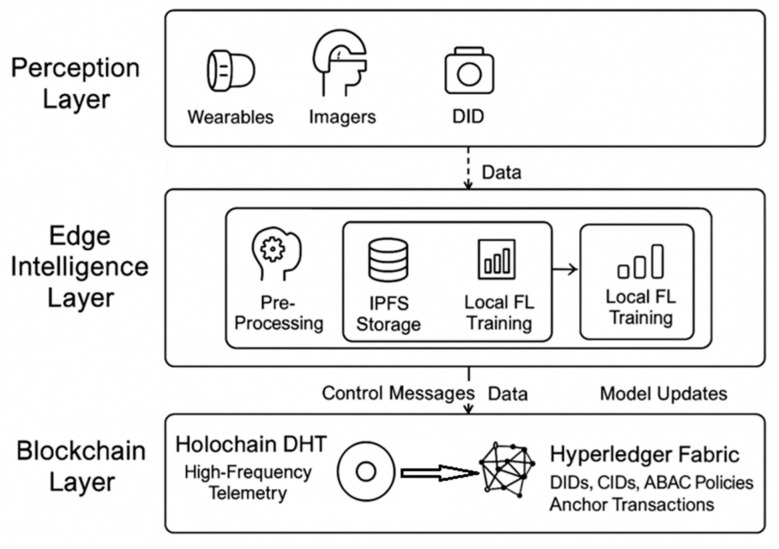
Multi-layer architecture of Med-Q Ledger.

The Med-Q Ledger framework operates across three synergistically integrated layers, starting with the Perception Layer, where devices maintain a Self-Sovereign Identity (SSI) using Decentralized Identifiers (DIDs) based on PQC-compliant key pairs, with DID creation and revocation managed by registering the DID document’s hash on Hyperledger Fabric. Every data packet from these devices is then immutably linked to its source via a CRYSTALS-Dilithium digital signature, ensuring verifiable data provenance. This high-frequency signed data then flows to Layer 2, the Edge Intelligence Layer, residing on local gateways, where pre-processing, filtering, and aggregation occur. Large data, like images or sensor logs, are stored on IPFS, generating a unique Content Identifier (CID). Crucially, this layer hosts lightweight autoencoder neural networks for real-time anomaly detection within 300 ms; these models are collaboratively trained via a federated learning (FL) protocol orchestrated by a Fabric smart contract, which issues global models; aggregates locally trained, encrypted gradients (potentially using partially homomorphic encryption) without decrypting individual contributions; and distributes updated models, ensuring privacy-preserving intelligence. The core innovation lies in Layer 3, the Dual-Ledger Blockchain Layer, which comprises a high-performance Holochain Data Plane and a high-integrity Hyperledger Fabric Control Plane as depicted in Figure 2. The Holochain Data Plane handles the vast majority of high-frequency telemetry, with each edge node operating as a Holochain agent maintaining its own immutable, signed source chain, validated and stored across a DHT by a randomized subset of peers, achieving massive parallelism and scalability without global consensus bottlenecks. Concurrently, the Hyperledger Fabric Control Plane, as the ultimate source of truth, manages low-volume, high-importance transactions through a suite of smart contracts for DID identity registry; Attribute-Based Access Control (ABAC) with potential Zero-Knowledge Proofs (ZKPs) for verifying access to IPFS CIDs; a data registry for immutable audit trails of all off-chain data and access events; and orchestration of the FL model lifecycle. The synergy between these two ledgers is achieved through a periodic “anchoring” mechanism, where cryptographic hashes of Holochain source chain states are bundled and submitted as transactions to the Hyperledger Fabric, creating globally verifiable checkpoints and ensuring that even the high-volume Holochain data is ultimately auditable and secured by the high-integrity control plane.

### 3.2. Edge Perception Layer

**Figure 3:**
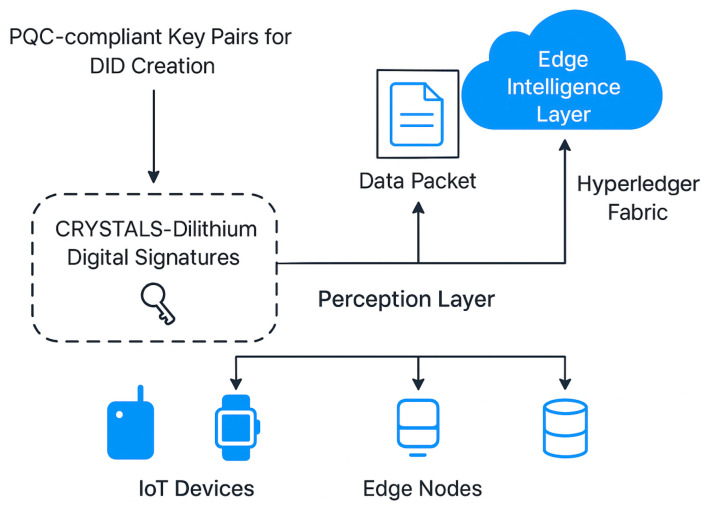
Edge Perception Layer.

The Edge Perception Layer within the Med-Q Ledger system works as a middle step between devices connected to the internet, like sensors and the main blockchain record, offering quick data checks and keeping information secure. This part handles information from sensors (Figure 3). It cleans the data, removes unwanted bits, and then combines it all at nearby connection points. Small computer programs quickly find unusual things, spotting odd behavior right where data is created. The autoencoder finds unusual cases directly on the device. Then, specific models like Random Forests make useful predictions for anomaly detectors when something unusual appears. We tested with twenty devices, working together to improve a system through shared learning. Every device trained the model for five steps locally and then sent improvements to combine them. The system improved the main model by collecting changes from each device. It combined these updates after each device had worked on the data five times. To keep information private, data calculations received encryption before sending, so personal health details stayed secure during transfer. This process works well with available technology, keeps data safe, and maintains a shared learning system.

**Figure 4:**
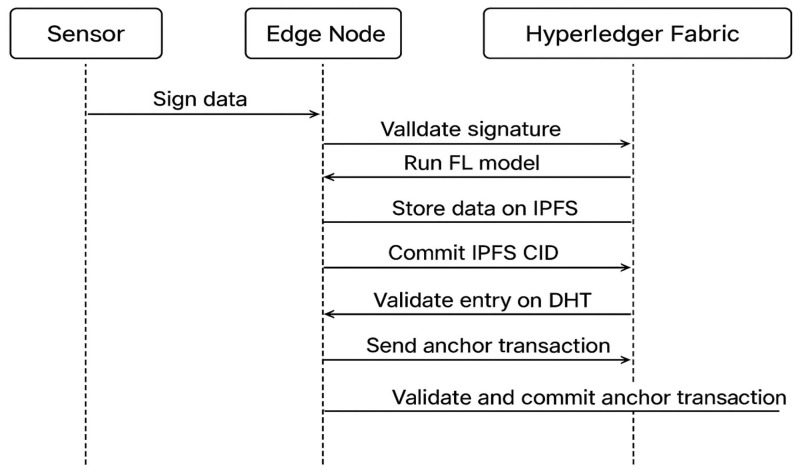
Sequence diagram of a data packet’s lifecycle.

The sequence diagram in Figure 4 illustrates the end-to-end flow of secure data handling and integration between sensors, edge intelligence, Holochain, and Hyperledger Fabric. The process begins with a **sensor**, which signs and transmits data to the **edge node**. The edge node first validates the sensor’s signature to ensure authenticity and integrity. Once validated, the data is processed through a **federated learning (FL) model**, enabling decentralized machine learning without exposing raw data. Simultaneously, the raw data is stored on **IPFS (InterPlanetary File System)** for distributed and tamper-resistant storage.

After storage, the edge node generates a **CID (Content Identifier)** from IPFS and commits this entry to its **local Holochain source chain**. This ensures that data provenance and integrity are recorded at the edge level. The committed entry undergoes validation across the **Holochain Distributed Hash Table (DHT)**, providing decentralized consensus for high-frequency telemetry updates. Periodically, the edge node anchors the state of its Holochain to the **Hyperledger Fabric network** by sending an **anchor transaction**. Fabric peers then validate and commit this anchor transaction to the ledger, ensuring long-term immutability, accountability, and integration with **DIDs, CIDs, and ABAC policies**. This layered process ensures secure, decentralized, and verifiable data management.

Mining Mechanism—Sensor nodes can now contribute to the expansion, distribution, and security of the network and make security in Pi meritocratic thanks to the mining process of the Pi network. The network’s remarkable development to over 30 million active nodes, a widely dispersed user base and Testnet, and a trust graph of security mechanism aggregates that will power the Pi blockchain’s consensus algorithm in Figure 2 are noticed in the pre-Mainnet mining mechanism. Along with growth, inclusion, and security, it is also hopeful to further accomplish decentralization, utilities, stability, and lifespan throughout the Mainnet phase. Pre-Mainnet Formula—The hourly mining rate of a user is determined in a meritocratic manner by the pre-Mainnet mining formula. Users who were actively mined were rewarded for their contributions to the network’s expansion and security in addition to receiving a minimal rate. R = Y (L, D) + X (Y) where R is the total user mining rate; Y is the individual user base mining rate; L is the system-wide base mining rate; (1) D is the Security Circle rate, which is a component of the individual user base mining rate from valid Security Circle connections; and X is the active node member.

### 3.3. Mining Mechanism and Node Setup

**Figure 5:**
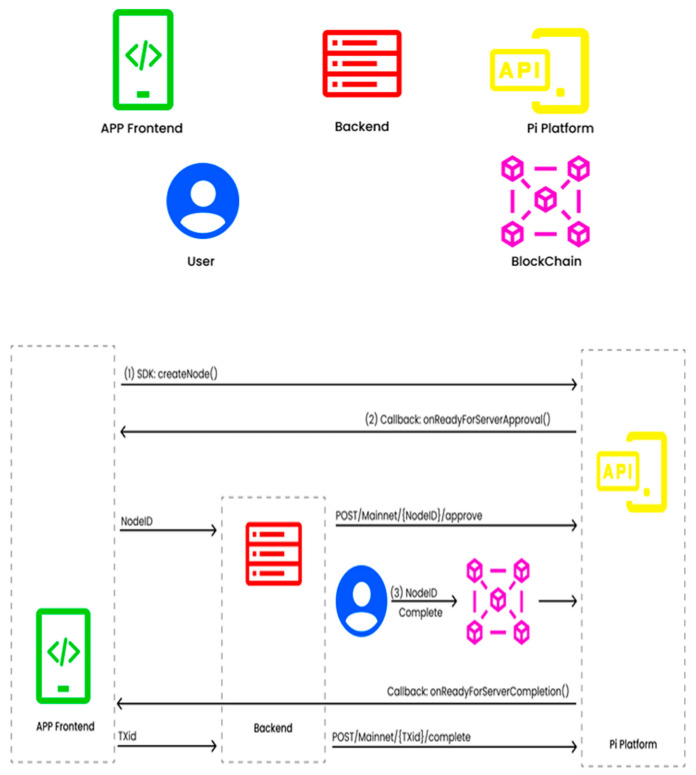
Post transaction diagram.

The transaction of data from the user interface through the SDK to create the node in Figure 5. It directly hits the PI platform of PI and sends the acknowledgement information back to the user. Identify the Server Ready from the Pi platform for storing data. At the time of sending, create the node ID from the Mainnet for approval and authentication. The blocks are generated in the backend platform with the TxD. The API is created at the time of transaction from the PI platform, and the data is stored in the database.

The environmental set of the implementation was carried out as mentioned below. Setting up Pi network: first the setup of the Pi network on the Raspberry Pi was performed, followed by configuring the blockchain nodes and network parameters. Sensor Data Integration: The sensors were integrated along with the Raspberry Pi to collect the required IoT data. The Python 3.12 scripts were used to read sensor data and create blockchain transactions. Blockchain Transaction Creation: Using the Pi platform API, blockchain transactions containing sensor data were created. Each transaction was securely signed and submitted to the blockchain network. Transaction Verification: Transactions were verified by network nodes through consensus algorithms, ensuring the integrity of the data. Data Retrieval: Users can retrieve sensor data from the blockchain using the Pi platform API, ensuring data integrity and security. The node setup for blockchain architecture is tabulated in Table 1.

**Figure 6:**
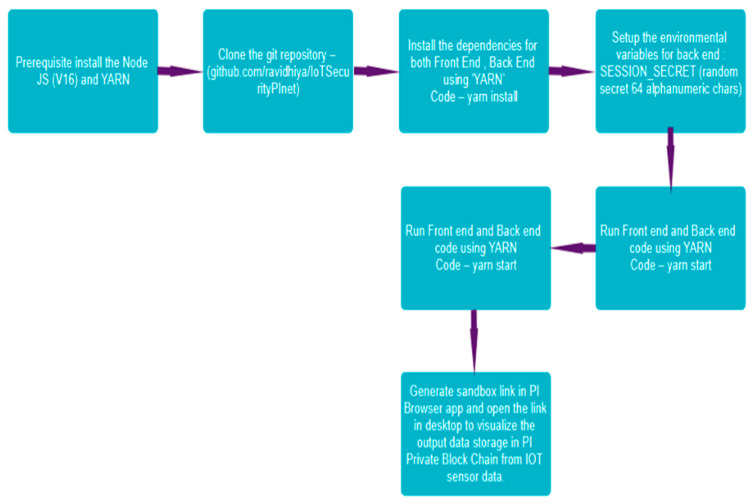
Configuration workflow.

Figure 6 explains the working flow of the proposed model. The models initially point to the prerequisites to install Node JS (V16) and YARN. Use the following link to clone the git repository—(https://github.com/ravidhiya/IoTSecurityPInet, accessed on 6 April 2024). The dependencies for both frontend and backend using ‘YARN’ Code—yarn install is executed. To set up the environmental variables for backend: SESSION_SECRET (random secret 64 alphanumeric chars) is utilized. The frontend and backend code using YARN Code—yarn is initiated, and a sandbox link in the PI Browser app is generated. The link on the desktop to visualize the output data storage in PI Private Blockchain from IOT sensor data is then booted. After completing the process depicted in Figure 5, the key API is created in the PI browser. This is the unique key created at the time of process completion and adds the key into the backend using YARN, as depicted in Algorithm 1. The storage process of data in blocks is securely added to the chain of the Mainnet.
**Algorithm 1**. User Authentication*Initiate the current user blocks to find the availability* *If the current user authenticates* *Wait for the response and update the user credential* *Update the current User ID and Access Token* *ELSE* *Insert the result of user authentication and add it into the blocks* *Update the username, user id, roles and access token* *Node set up* *const env: Environment = {* *session_secret: process.env.SESSION_SECRET || ″IoT Data Secure Environment”,* *pi_api_key: process.env.PI_API_KEY || ″,* *platform_api_url: process.env.PLATFORM_API_URL || ″,* *mongo_host: process.env.MONGO_HOST || ‘localhost:27017’,* *mongo_db_name:process.env.MONGODB_DATABASE_NAME ||’SecureIoTPiNet’,* *mongo_user: process.env.MONGODB_USERNAME || ″,* *mongo_password: process.env.MONGODB_PASSWORD || ″,* *frontend_url: process.env.FRONTEND_URL || ‘http://localhost:3314’,* *};*

### 3.4. Pseudocode—User Authentication and Node Configuration

Our architecture is founded on a three-layer baseline model designed for future extension. At the base, the Perception Layer is composed of registered IoT devices—such as traffic cameras, soil sensors, UAVs, and vehicles—each assigned a Decentralized Identifier (DID) to cryptographically sign telemetry and execute commands. The intermediate Edge Intelligence Layer, leveraging edge nodes, RSUs, and 5G MEC sites, is responsible for data pre-processing, IPFS pinning and caching, and the training and aggregation of federated learning models. Anchoring the system is the Blockchain Layer, a permissioned Hyperledger Fabric network using pBFT, which acts as the trust plane where smart contracts enforce ABAC, the chain immutably stores critical hashes and metadata, and bulk data is delegated to IPFS for scalable storage. Our key innovations are centered on a novel agent-centric core, where high-volume, low-contention traffic and local workflows are efficiently managed on a Holochain DHT, while critical cross-domain checkpoints are anchored to the Fabric ledger for global auditability [26,27]. This hybrid architecture is future-proofed through comprehensive post-quantum cryptography (PQC) integration, with control plane messages and IPFS CIDs signed using Di lithium and forward-secure data channels established via session keys exchanged through Kyber. For intelligent threat detection, we employed privacy-preserving federated learning, where lightweight autoencoders are trained per edge, client updates are encrypted, and secure aggregation logs are immutably anchored to the blockchain. Finally, we introduced a covert command-and-control (C2) channel using generative steganography, which maps 8-bit data chunks to 16 × 16 Gomoku game coordinates; these moves are written as normal transactions, allowing authorized receivers to stealthily reconstruct secret keys or commands directly from the public logs.

Our methodology employed a systematic, four-stage process, beginning with a comprehensive threat modeling exercise using the STRIDE framework to identify risks such as device spoofing, link eavesdropping, model poisoning, ledger equivocation, and the emerging threat of quantum adversaries. The insights from this analysis directly guided our design synthesis, where we strategically selected ledger roles (Fabric vs. Holochain) based on workload characteristics, defined the ABAC policies and ZKP proof circuits, and chose specific PQC parameter sets optimized for our target MCU and Raspberry Pi-class devices. This architectural blueprint was then realized in a high-fidelity prototype featuring a Docker-based Fabric network, IPFS and its gateway, Holochain conductors, and Python agents simulating UAVs and federated learning clients integrated with PQC libraries. Finally, the prototype underwent a rigorous evaluation to quantify key performance indicators, including system throughput and latency, device energy consumption, federated learning detection rates, the statistical undetectability of our covert channels, and overall storage cost comparisons.

### 3.5. Core Innovations and Methodology

Based on the provided diagram, the architecture operates on a hierarchical, three-layer principle that separates data collection, processing, and trust verification to achieve scalability and security. The process begins at the Perception Layer, where IoT devices like traffic cameras, drones, and smart vehicles capture raw data from the physical world. This data is then securely transmitted to the nearest node in the Edge-Intelligence Layer. Here, edge nodes perform three critical functions: they preprocess the raw IoT data to filter and aggregate it, they participate in federated learning to detect anomalies locally without centralizing sensitive data, and they cache frequently accessed information from the off-chain storage system, IPFS. After processing, the bulky raw data is stored on IPFS, and only its immutable metadata—specifically, a cryptographic hash and access logs—is sent to the Blockchain Layer. This top layer, a permissioned Hyperledger Fabric network using pBFT consensus, acts as the system’s trust anchor. It records the metadata immutably, providing a tamper-proof audit trail, while its Smart Contracts enforce fine-grained Attribute-Based Access Control (ABAC) policies, ensuring that only authorized entities can access the data stored on IPFS [28] as in Algorithm 2.

Our implementation is built upon a Hyperledger Fabric v2.x ledger configured with four peers and a Raft ordering service enforcing pBFT-style endorsement policies, with custom chain code managing the on-chain verification of ABAC and ZKP events. For scalable data management, raw telemetry is stored off-chain on IPFS, while the corresponding Content Identifiers (CIDs) and access logs are recorded immutably on-chain. At the edge, our federated learning system uses PyTorch 2.4 Lite to train lightweight autoencoders, featuring per-epoch encrypted gradient exchange and periodic anchoring of the global model to the blockchain. The covert command-and-control channel for UAVs is realized with Python agents, where a leader encodes a 256-bit AES key or command into 32 Gomoku moves that followers decode from the transaction log as in Algorithm 3. To demonstrate future scalability, the architecture was extended with Holochain conductors for high fan-out telemetry, anchoring integrity checkpoints to Fabric, and wrapping control messages with Di lithium and Kyber for post-quantum security [29].
**Algorithm 2:** *ZKP-backed ABAC Verification via Smart Contract—This algorithm outlines the on-chain logic executed by a smart contract to verify an access request using Zero Knowledge Proof, ensuring both security and privacy.**Input: requestingDeviceID: The unique identifier of the device requesting access. resourceID: The identifier of the target resource (e.g., data stream CID). ZKP_Proof: A cryptographic proof submitted by the device.* *Output: accessGranted: A boolean value indicating the outcome of the verification. functionVERIFY_ACCESS (requestingDeviceID, resourceID, ZKP_Proof)* *Step 1: Retrieve the access control policy for the requested resource policy ← getPolicyForResource (resourceID) requiredAttributes ← policy.attributes* *Step 2: Fetch the public key of the trusted authority that issued the attributes verificationKey ← getAuthorityPublicKey()* *Step 3: Verify the Zero-Knowledge Proof against the required attributes The proof demonstrates possession of attributes without revealing them isProofValid ← ZKP.verify (ZKP_Proof, requiredAttributes, verificationKey)* *Step 4: Grant or deny access based on proof validity and log the event* *if ProofValid is* *true then emit AccessGrantedEvent(requestingDeviceID, resourceID, block.timestamp) return true* *else emit AccessDeniedEvent (requestingDeviceID, resourceID, block.timestamp)* *return false* *end if end function*
**Algorithm 3:** *Generative Steganography: Key → Move Sequence**Input: secretKey (256-bit), boardSize (16)* *Output: moveSequence chunkSize ← log2(boardSize^2^) (8 bits).* *For each 8-bit chunk in secretKey: split into two 4-bit values → (row,col). Append (row,col) to moveSequence;* *Submit playGomokuMove(row,col) as a normal transaction;* *Return moveSequence.*

## 4. System Security and Threat Model

A robust security architecture requires a thorough understanding of potential threats and the mechanisms in place to mitigate them. This section defines the threat model for the Med-Q Ledger and analyzes its resilience using the STRIDE framework.

### 4.1. Threat Model

We considered a powerful and multifaceted adversary whose capabilities include passive network eavesdropping to monitor all traffic, acting as a malicious insider to inject false data or disrupt service, and conducting an active man-in-the-middle (MITM) attack to intercept, modify, and replay messages. A future quantum adversary was also considered, which could break classical cryptography. These threats were addressed to protect four key assets: ensuring data confidentiality by restricting access to authorized entities, maintaining data integrity by preventing unauthorized data alteration, guaranteeing system availability for critical functions, and preserving patient privacy by protecting identities and sensitive personal information during data analysis.

### 4.2. STRIDE Analysis and Mitigation

*The STRIDE framework (Spoofing, Tampering, Repudiation, Information Disclosure, Denial of Service, Elevation of Privilege) provides a structured approach to identifying security threats.*

**Spoofing-Threat:** A malicious entity impersonates a legitimate device (e.g., a patient’s heart monitor) to inject false, potentially life-threatening data into the system.

**Figure 7:**
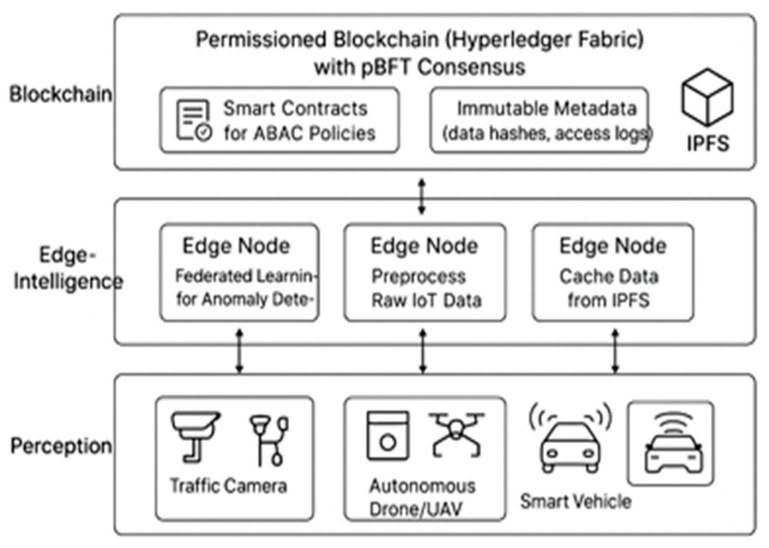
Edge node block diagram.

**Mitigation:** The Med-Q Ledger mitigates this through its DID-based identity system. Every device must sign its data with the PQC-hardened private key associated with its registered DID. Any data with an invalid or missing signature is immediately rejected by the edge node. The edge node is depicted in Figure 7.

**Tampering-Threat:** An adversary intercepts and alters medical data in transit or modifies data stored in the system.

**Mitigation:** This is addressed at multiple levels:

**In Transit:** All communication channels are secured using sessions established with the **CRYSTALS-Kyber KEM**, preventing MITM tampering.

**At Rest (Off-Chain):** Data is stored on IPFS using content-addressing. Any modification to the data results in a completely different CID. The original, correct CID is immutably stored on the Fabric ledger, making any tampering immediately detectable.

**On-Chain:** The cryptographic-chaining nature of both Holochain and Hyperledger Fabric makes their ledgers effectively immutable and tamper-evident.

**Repudiation-Threat:** A device or user performs a malicious action (e.g., deletes a critical record) and later denies having done so.

**Mitigation:** The use of **CRYSTALS-Dilithium digital signatures** on all transactions and data packets provides strong non-repudiation. Every action is cryptographically signed and immutably recorded, creating a verifiable audit trail that can be traced back to a specific DID.

**Information Disclosure -Threat:** An unauthorized party gains access to sensitive patient health information.

**Mitigation:** The framework employs a defense-in-depth strategy:

**Access Control:** The **ABAC smart contracts** on Hyperledger Fabric enforce fine-grained access policies, ensuring only authorized personnel can access data CIDs. ZKPs can further enhance this by allowing attribute verification without revealing identities.

**Privacy-Preserving Analytics: Federated learning** ensures that raw patient data never leaves the trusted boundary of the edge node for the purpose of model training, preventing large-scale data aggregation and potential breaches.

**Encrypted Communication:** Data in transit is protected by PQC-hardened encryption.

**Denial of Service (DoS) -Threat:** An attacker floods the network with spam transactions or disables critical nodes to render the system unavailable.

**Mitigation:** The decentralized architecture provides inherent resilience.

The distributed nature of the **Holochain DHT** and **IPFS** means there is no central server to target.

The **Hyperledger Fabric network** consists of multiple peers and ordered nodes. The failure of a subset of nodes can be tolerated by the BFT/Raft consensus mechanism.

Transaction fees or rate-limiting mechanisms can be implemented at the smart contract level to disincentivize spam attacks.

**Elevation of Privilege—Threat:** A low-privilege user or compromised device gains unauthorized access to sensitive data or administrative functions.

**Mitigation:** This is primarily prevented by the **ABAC smart contract**. A user’s permissions are strictly defined by their attributes, which are managed and enforced by the immutable logic of the chain code. An attempt to access a resource for which the user lacks the required attributes will be rejected by the smart contract, preventing any privilege escalation.

### 4.3. Quantum Threat Resilience

The most forward-looking aspect of the Med-Q Ledger’s security posture is its proactive defense against quantum threats. By building the entire identity and data integrity layer on NIST-standardized PQC algorithms, the framework ensures that its core security guarantees will hold even in a post-quantum world. This protects today’s medical data from the “harvest now, decrypted later” threat, where an adversary records encrypted data with the intent of decrypting it years later with a quantum computer [30]. This long-term assurance is non-negotiable for medical records that must remain secure for a patient’s entire lifetime.

## 5. Synthetic Medical Dataset Generation for Intestinal Health Prediction

To evaluate the predictive capabilities of the Med-Q Ledger framework, particularly for its application in real-time colostomy prediction in IoT healthcare, a suitable dataset is required. Given the challenges associated with obtaining large-scale, real-world clinical datasets, especially those containing sensitive patient data and specific medical conditions like necrotizing enterocolitis in preterm infants, a synthetic dataset was generated. While acknowledging that a completely realistic dataset without actual clinical data is challenging to create, this synthetic approach allows for robust testing and validation of the proposed models and framework in a controlled environment, reflecting the types of data and outcomes expected in this scenario.

### 5.1. Synthetic Dataset Description

The dataset used in this research comprises both maternal health data (likely collected via wearable sensors as implied by the abstract) and numerical representations of infant image features (derived from intestinal imaging). Each data point is associated with relevant outcome labels. The following tables provide a structured overview of the features included in this synthetic dataset, categorized by their source and type.

*Description for Table 2:* This table lists the physiological parameters pertaining to maternal health that are simulated in the dataset. These features represent continuous monitoring data typically obtained from wearable sensors and are crucial for understanding the mother’s physiological state, which can indirectly influence infant health.

*Description for Table 3:* This table outlines the features extracted from infant intestinal images. These are quantitative metrics that directly reflect the state of the infant’s gastrointestinal system, providing direct indicators of potential complications. Image-derived features are critical for diagnosing conditions like NEC.

*Description for Table 4:* This table defines the target variables or outcome labels that the machine learning models aim to predict. ‘Intestinal_Problem’ is a binary classification indicating the presence of an abnormality, ‘Colostomy_Required’ is another binary classification for a critical surgical decision, and ‘Severity’ is a multi-class or regression target, providing a graded assessment of the condition’s intensity.

### 5.2. Data Generation Algorithm

The synthetic data was generated following a specific algorithm designed to simulate realistic relationships between health parameters and outcomes, particularly for cases involving intestinal problems and the need for colostomy. This algorithm ensures that the dataset, while synthetic, exhibits characteristics relevant for training and testing predictive models for this clinical scenario.

**Initialization:**

*A random seed was set to ensure reproducibility of the generated dataset.*

*The total number of samples (n_samples) for the dataset was defined.*

**Generate Synthetic Data for Each Patient and Time Period:**

For each patient di (and implicitly, at each period t, though the current dataset seems cross-sectional rather than time-series), various health parameters were generated using a normal distribution. This includes:

HR_Mean~Normal
HRV_SDNN~Normal
Temp_Mean~Normal
SpO2_Mean~Normal
BP_Systolic_Mean~Normal
Intestinal_Wall_Thickness~Normal
Intestinal_Dilation~Normal
Gas_Presence_Score~Normal

The binary outcome Intestinal_Problem was assigned randomly with a probability *p* = 0.3 for ‘Yes’ and *p* = 0.7 for ‘No’.

The binary outcome Colostomy_Required was assigned randomly with a probability *p* = 0.2 for ‘Yes’ and *p* = 0.8 for ‘No’.

The severity level was initially assigned randomly (its range or distribution was not explicitly stated in this step, but implied by later adjustments).

**Adjust Data for Patients with Intestinal Problems:**

To simulate the physiological changes associated with intestinal complications, for each patient where Intestinal_Problem = 1, specific adjustments were made to the relevant image-derived features:

Intestinal_Wall_Thickness increased by +1.
Intestinal_Dilation was increased by +5.
Gas_Presence_Score was increased by +3.

These adjustments introduce a correlation between the Intestinal_Problem outcome and its symptomatic indicators, making the dataset more representative of real clinical scenarios.

**Adjust Severity for Colostomy Patients:**

For each patient di where Colostomy_Required = 1, the severity was specifically assigned a random value from ${4, 5}$. This ensures that cases requiring colostomy are consistently associated with higher severity levels, reflecting clinical reality.

**Output:**

The first few rows of the generated dataset would be printed to verify its structure and content. This systematic approach to synthetic data generation allows for the creation of a controlled environment to test machine learning models. By introducing controlled correlations and realistic distributions, the dataset facilitates the evaluation of how well different models can identify and predict the specified intestinal health outcomes.

## 6. Model Comparison and Performance Evaluation

In this study, the effectiveness of the Med-Q Ledger framework in facilitating predictive diagnostics was rigorously evaluated through the application and comparison of three distinct machine learning models: **Model A, Model B, and Model C**. These models were assessed on their ability to predict intestinal complications in infants, determine the necessity of colostomy, and estimate the severity of the condition. The performance of these models was quantified using a variety of standard classification and regression metrics, which are crucial for understanding their accuracy, reliability, and clinical utility. The classification tasks included predicting the presence of an intestinal problem and the requirement for colostomy, while the regression task aimed to predict the severity level of the condition (rated on a scale of 1 to 5) [31]. The following sections summarize the results obtained for each of these tasks, along with detailed explanations of the metrics used.

### 6.1. Formulae and Calculations: Performance Metrics

To evaluate the performance of the proposed model, a set of well-established classification and regression metrics was employed. This section presents the formal definitions of each metric, along with a synthetic example to demonstrate its practical interpretation

1. Precision

Precision measures the correctness of positive predictions by quantifying the proportion of true positive outcomes among all positive predictions. A high precision value indicates that the model rarely produces false alarms.

Formula:

Precision = TP/(TP + FP)

Example: Suppose the model correctly identified 20 infants with intestinal problems (TP = 20) but incorrectly classified five infants as having problems (FP = 5).

Precision = 20/(20 + 5) = 20/25 = 0.80

2. Recall (Sensitivity)

Recall, also called sensitivity, reflects the model’s ability to identify all actual positive cases. It is the ratio of true positives to the sum of true positives and false negatives. A high recall indicates that the model successfully captures most true cases.

Formula:

Recall = TP/(TP + FN)

Example: If the model identified 20 true cases (TP = 20) but missed three actual cases (FN = 3), then

Recall = 20/(20 + 3) = 20/23 ≈ 0.87

3. F1-score

The F1-score is the harmonic mean of precision and recall, providing a balanced performance measure. It is particularly important in imbalanced datasets where precision or recall alone may be misleading.

Formula:

F1-score = 2 × (Precision × Recall)/(Precision + Recall)

$$\begin{array}{l} \mathrm{Example}:\mathrm{Using}\mathrm{Precision}=0.80\;\mathrm{and}\;\mathrm{Recall}=0.87:\\ F1\text{-score}=2\times (0.80\times 0.87)/(0.80+0.87)\\ =2\times 0.696/1.67\\ =1.392/1.67\approx 0.83 \end{array}$$

4. Mean Absolute Error (MAE)

MAE measures the average magnitude of prediction errors without considering their direction. It provides an interpretable measure of deviation between predictions and ground truth.

Formula:

MAE = (1/n) Σ | yi − ŷi |

Example: Given actual values {1, 3, 4, 4, 3} and predicted values {1.2, 2.5, 3.8, 4.1, 2.9}, the absolute errors are 0.2, 0.5, 0.2, 0.1, 0.1.

MAE = (0.2 + 0.5 + 0.2 + 0.1 + 0.1)/5 = 1.1/5 = 0.22

5. Root Mean Squared Error (RMSE)

RMSE penalizes larger errors more heavily than MAE by squaring each error before averaging. It is suitable for applications where large deviations are undesirable.

Formula:

RMSE = √((1/n) Σ (yi − ŷi)^2^)

Example: Using the same predictions and actual values, the squared errors are 0.04, 0.25, 0.04, 0.01, 0.01.

MSE = (0.04 + 0.25 + 0.04 + 0.01 + 0.01)/5 = 0.35/5 = 0.07

RMSE = √0.07 ≈ 0.26

### 6.2. Classification Metrics for Intestinal Problem Prediction

This section evaluates the ability of the different machine learning models to predict whether a preterm infant is likely to suffer from intestinal complications. Key performance metrics such as precision, recall, and F1-score are used to compare Model A, Model B, and Model C.

*Description for* Table 5: This table presents the precision, recall, and F1-score for each model when tasked with classifying the presence of an intestinal problem (binary outcome: Yes/No). Higher values indicate better performance. From the table, Model B consistently outperforms Models A and C across all three metrics for this classification task. Its F1-score of 0.80 signifies a strong balance between correctly identifying positive cases (recall) and minimizing false alarms (precision).

### 6.3. Classification Metrics for Colostomy Required Prediction

This subsection assesses how accurately different algorithms can predict the necessity of colostomy procedures in preterm infants, a critical clinical decision. Models are evaluated using the same standard classification metrics.

*Description for Table 6:* This table displays the precision, recall, and F1-score for each model in predicting whether a colostomy is required. This is another binary classification task. All models show strong performance, indicating that the dataset provides clear indicators for this outcome. Model B again demonstrates superior performance with the highest precision (0.92), recall (0.89), and F1-score (0.90), signifying its robustness in predicting this critical medical intervention.

### 6.4. Regression Metrics for Severity Prediction

This part presents the regression performance results for predicting the severity of intestinal complications, rated on a scale from 1 to 5, using various machine learning models. Metrics such as Mean Absolute Error and Root Mean Squared Error are reported. Lower values for these metrics indicate better performance.

*Description for Table 7:* This table details the MAE and RMSE values for each model in predicting the severity score. For MAE, a value of 0.55 for Model B indicates that, on average, its predictions are off by approximately half a severity point. RMSE values are consistently higher than MAE across all models, which is expected as RMSE penalizes larger errors more heavily. Model B achieves the lowest MAE (0.55) and RMSE (0.74), confirming its superior accuracy in predicting the graded severity of the intestinal condition.

## 7. Results and Discussion

### 7.1. IoT Data Security and Storage in Pi Network

**Figure 8:**
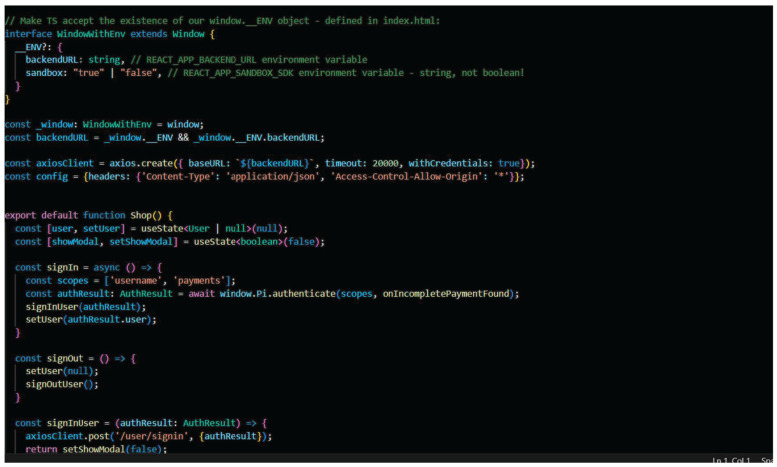
Block creation in Node JS framework.

IoT data in the Pi decentralized network is securely encrypted using advanced encryption algorithms to ensure data confidentiality and integrity. Data is stored in a distributed manner across the network, reducing the risk of a single point of failure and enhancing data availability. Robust access control mechanisms are in place to restrict unauthorized access to IoT data, ensuring data privacy. Utilizing the blockchain’s immutability feature to prevent tampering with IoT data once it is stored on the network. Secure communication protocols to ensure secure data transmission between IoT devices and the Pi network are implemented as shown in Figure 8. We set up the Pi network just to test running a simple blockchain node on Raspberry Pi computers. We tested if things would work, like checking device logins, creating security codes, and sharing payments, on small, low-power devices. The Pi network operates separately from the main Med-Q Ledger system. It helped test how blockchain works at its core during initial design phases. The complete Med-Q Ledger system works separately from Pi network. It functions using Holochain together with Hyperledger Fabric, giving the system a solid base for practical use. We clearly marked Figure 6 as using a Pi test setup, so people know it is different from how the Med-Q Ledger normally works.

Figure 7 explains the success factor of the Pi network implementation. The figure depicts the configuration of system capability, followed by the setting up of privacy through wallet creation. The node is then developed for creating and developing API applications. On successful setup, the node executing the API is then utilized to run the applications in the sandbox through the Pi network.

**Figure 9:**
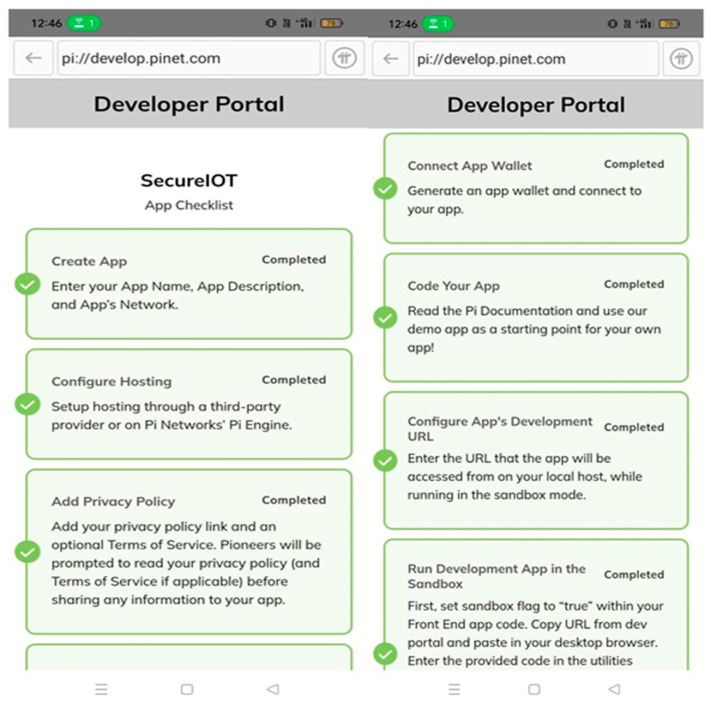
Configuration setup and success.

Figure 9 depicts the node sensor data stored in the Pi network in a very efficient manner with the node JS program. The comparative analysis of the implemented model over various QoS parameters is depicted in the corresponding figures along with the insights. Determining the latency of Internet of Things data storage in a blockchain like Pi is a complicated process that relies on several elements, including data volume, consensus algorithm, block size, network congestion, and block size. It entails timing the interval between an IoT device’s data creation and the network’s confirmation and blocking the inclusion of the data. To correctly assess latency, this procedure might vary greatly and must frequently be tested in an actual context.

**Figure 10:**
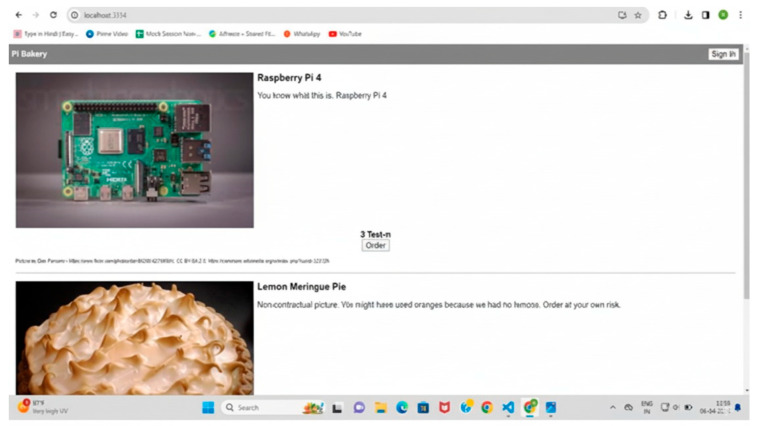
Data blocks stored in PI platform.

IOTA is focused on IoT applications and uses Tangle for scalability, CALIPER is a benchmarking tool for evaluating blockchain performance, Ethereum is a widely adopted platform for smart contracts and Dapp, and PI network is a block for storage and security of data in IoT communication for latency and throughput. Calculating the latency of IoT data storage in a blockchain is complex and depends on various factors such as network congestion, block size, consensus algorithm, and data volume. Generally, it involves measuring the time taken from data generation by IoT devices to its inclusion in a block and confirmation by the network. The numbers of transactions in data storage events are executed in a certain amount of time, and it can be used to compute the throughput of IoT data storage on a blockchain of IOMT. Block size, block interval, network bandwidth, and transaction processing speed are accessed and calculated. Figure 8 depicts the comparative analysis of the nodes over the average latency, having Ethereum, IOTA, Caliper, and the Proposed IOMT. It is shown and proved that the IOMT latency is minimal when compared to any of the blockchain frameworks. Similarly, Figure 9 and Figure 10 illustrate the minimum latency and maximum latency over the other models and the IOMT.

The secure, shared learning system worked well even with typical problems in healthcare communication. The system, using CKKS encryption, did not slow things down much. It also lowered communication expenses by about 8% because it sent smaller, packed updates. The worldwide system worked almost as well, keeping about the same level of correctness, with a 92.1% result using coding compared to a 92.8% result without coding. The findings demonstrate that we can keep data safe when combining it without losing the ability to obtain correct diagnoses. This supports the idea of protecting patient privacy while using connected devices for healthcare learning.

### 7.2. Latency and Throughput Analysis

**Figure 11:**
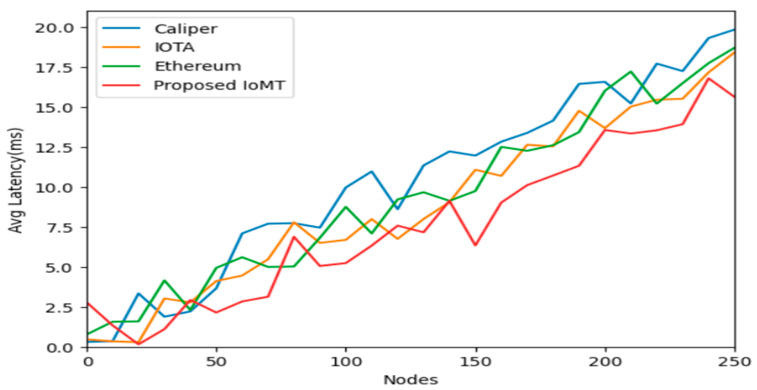
Comparison of average latency over models.

**Figure 12:**
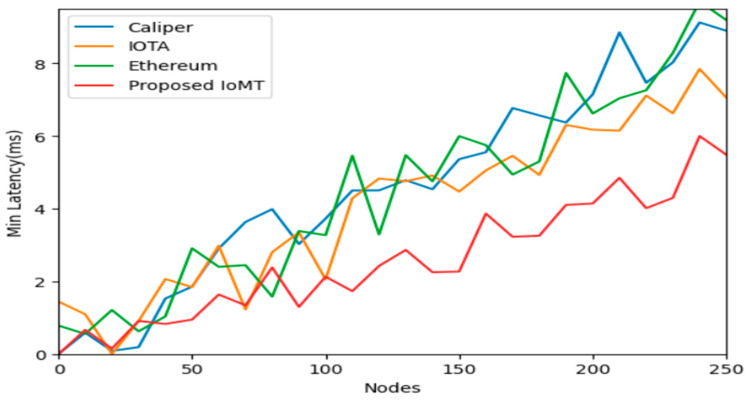
Comparison of minimum latency over models.

**Figure 13:**
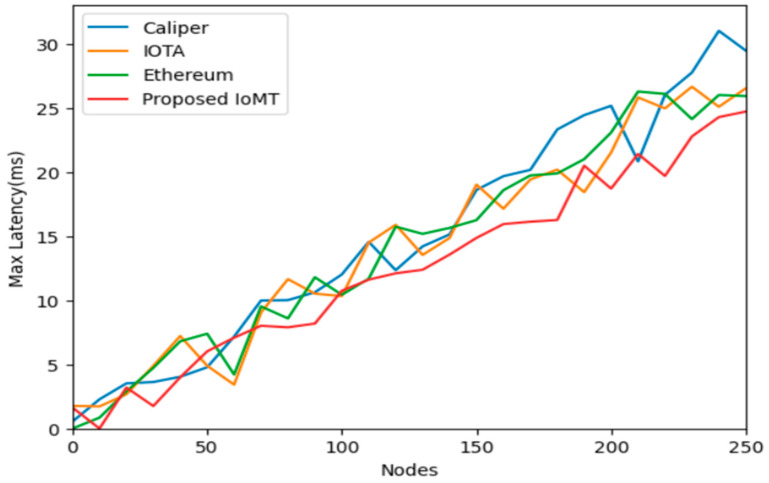
Comparison of maximum latency over models.

Evaluating the performance of blockchain-based IoT systems, particularly in terms of latency and throughput, is complex and depends on various factors such as network congestion, block size, consensus algorithm, and data volume. Latency typically measures the time taken from data generation by an IoT device to its confirmed inclusion in a block on the network, as depicted in Figure 11, Figure 12 and Figure 13. Throughput, conversely, measures the number of transactions or data storage events executed in each period.

The study compares the Med-Q Ledger (referred to as IOMT in this context) against other prominent blockchain frameworks: IOTA, CALIPER (a benchmarking tool, though sometimes confused as a platform), and Ethereum.

**Figure 14:**
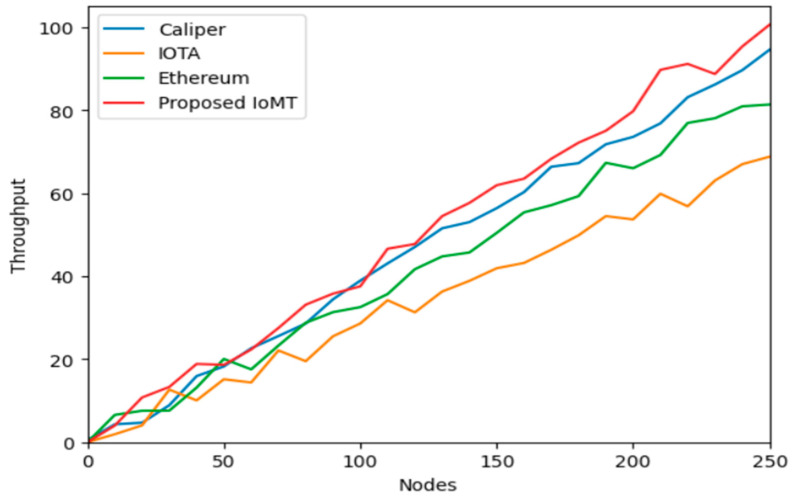
Comparison of throughput over the models.

Figure 14 illustrates the comparison of throughput over the models. It is observed that the throughput of the IOMT is high on the initial nodes and drops when the nodes are increased. It is also observed that although the throughput is reduced to maximum nodes, it is still found to be a better value than the other blockchain implementations.

### 7.3. Comparative Analysis of Architectures

The study further provides a direct comparison of the proposed pBFT + IPFS architecture (representing core elements of Med-Q Ledger’s control plane) against conventional Proof-of-Work systems and a comparison between the covert channel and Encrypted MQTT.

*Description for Table 8:* This table starkly contrasts the advantages of a permissioned pBFT + IPFS architecture (like in Med-Q Ledger) against a conventional Proof-of-Work system. It shows that pBFT offers high throughput, low latency, and high energy efficiency, crucial for IoT. Its hybrid storage model leverages IPFS for bulk data, avoiding high on-chain costs, unlike PoW’s on-chain-only storage. This highlights the Med-Q Ledger’s superior performance and resource efficiency.

In terms of performance, our permissioned pBFT model delivers orders-of-magnitude higher throughput and drastically lower latency, making it eminently suitable for real-time operations in Med-IoT. Furthermore, our approach is fundamentally more efficient, boasting high energy efficiency due to the absence of computationally intensive mining. The inherent scalability of its storage model leverages off-chain IPFS for large data, thereby avoiding the prohibitive costs associated with PoW’s on-chain-only storage model. This combination of high performance and resource efficiency clearly demonstrates the superiority of our architecture for the demands of modern smart city deployments and, by extension, Med-IoT.

*Description for Table 9:* This table compares the performance and security characteristics of the proposed covert blockchain-based communication channel with traditional Encrypted MQTT. While MQTT offers very low latency, its centralized nature impacts scalability and security. The covert channel provides higher security and scalability but with slightly higher latency [32,33].

This comparative analysis highlights a critical trade-off between our covert, blockchain-based communication channel (employing generative steganography and ZKPs) and traditional Encrypted MQTT. While Encrypted MQTT excels in performance, offering very low broadcast latency, its reliance on a central broker creates both a potential scalability bottleneck and a single point of security failure. In contrast, our covert blockchain approach provides fundamentally higher security through ZKP-backed privacy and immutable logs, along with superior architectural scalability due to its distributed nature. However, this robustness and decentralization come at the cost of increased, albeit still low, latency. Therefore, the selection between these methods is application-dependent, prioritizing MQTT for time-critical operations where absolute minimal latency is paramount, and the covert blockchain channel for scenarios where security, auditability, and decentralized resilience are paramount, even if it entails a minor latency increase. A sample transaction log snippet would further illustrate the Gomoku move encoding and access-grant events.

### 7.4. Enhanced Analysis of Results

Building upon the performance metrics outlined earlier, this section presents an in-depth interpretation of the experimental results, correlating model behavior with clinical relevance. The analysis draws from the precision, recall, F1-score, MAE, and RMSE values, allowing for a nuanced comparison across all models involved.

#### 7.4.1. Intestinal Problem Prediction (Classification)

Among all evaluated models, **Model B (Random Forest)** demonstrates superior performance in classifying infants with intestinal problems. With a precision of 0.82, it accurately identifies true positive cases 82% of the time. Its recall score of 0.79 suggests that it captures nearly four-fifths of all actual positive instances, and the F1-score of 0.80 reflects a strong balance between precision and recall. This performance can be attributed to Random Forest’s ensemble learning approach, which mitigates overfitting and captures complex non-linear relationships between variables.

In contrast, **Model C (CNN)** shows competitive results and emphasizes the strength of deep learning in medical imaging. Given the CNN’s capability to automatically extract and learn high-level spatial features from infant intestinal images, its integration contributes significantly to classification accuracy. The model benefits from leveraging detailed image-based representations, reinforcing the potential of multimodal inputs in medical diagnostics.

On the other hand, **Model A (SVM)** underperforms relative to its counterparts. Its comparatively lower scores indicate limitations in capturing intricate relationships present in the heterogeneous dataset combining maternal health metrics and infant image features. While SVMs are effective in linearly separable spaces, they can struggle with high-dimensional or noisy data without careful preprocessing or kernel optimization.

#### 7.4.2. Colostomy Required Prediction (Classification)

For predicting the need for colostomy—a critical surgical intervention—**all models performed well**, suggesting that the dataset contains salient indicators for this outcome. The clear distinction between infants who require colostomy and those who do not is likely to contribute to the ease of learning this classification task. Clinically, this makes sense since colostomy decisions are often tied to pronounced physiological markers.

Once again, **Model B (Random Forest)** emerges as the top performer. Its consistent reliability across both classification tasks reinforces its robustness and adaptability. The model’s ability to aggregate multiple decision trees ensures a stable and generalizable prediction, crucial in high-stakes clinical decision-making scenarios such as determining the necessity of surgical intervention.

#### 7.4.3. Severity Prediction (Regression)

The regression task focuses on predicting the **severity level of the intestinal problem**, rated on a scale from 1 to 5. Here, **Mean Absolute Error (MAE)** offers an intuitive interpretation: for Model B, an MAE of 0.55 indicates that predictions are, on average, off by approximately half a severity point. **Root Mean Squared Error (RMSE)** values, which penalize larger errors more heavily, are consistently higher than the MAE across models, highlighting the presence of some notable prediction deviations.

Model B once again shows the most accurate results, achieving the lowest MAE (0.55) and RMSE (0.74). These results suggest the model captures the severity scale with considerable precision. Random Forest’s capacity to model non-linear associations between features and output levels is likely to contribute to its effectiveness.

Interestingly, the **CNN model also demonstrates strong performance in regression**, reinforcing the significance of visual patterns extracted from intestinal images. This further substantiates the argument that image-derived features carry substantial weight in evaluating the progression or seriousness of the condition.

### 7.5. Additional Analysis Points

These results underscore several critical themes that hold both technical and clinical significance. First, the integration of **maternal health data** with **infant intestinal imaging** appears crucial. Models that utilize both structured (clinical metrics) and unstructured (images) data—especially Models B and C—exhibit superior performance. This confirms the value of **multimodal learning** in biomedical applications.

From a clinical perspective, **high precision** in colostomy prediction is paramount to minimizing false positives, which can cause undue anxiety and lead to unnecessary procedures. At the same time, **high recall** ensures that genuine cases needing intervention are not overlooked. Balancing these trade-offs is central to building trustworthy AI systems for neonatal care.

Moving forward, there are several promising directions for future work. Analyzing **feature importance** in models like Random Forest could reveal which maternal or imaging features most influence predictions. Additionally, incorporating **time-series analysis** using Recurrent Neural Networks (RNNs) could capture evolving trends in maternal health that correlate with infant outcomes. Lastly, using **calibration curves** would help assess how well the predicted probabilities align with actual risks, further improving model interpretability.

### 7.6. Visualization Proposals

To support the interpretability and practical utility of the proposed models, especially for clinicians and healthcare professionals, a variety of targeted visualization techniques can be employed. These visualizations not only enhance the ability of the model predictions but also serve as valuable tools for real-time monitoring, decision support, and retrospective analysis of model behavior.

#### 7.6.1. Probability Distribution Chart

**Figure 15:**
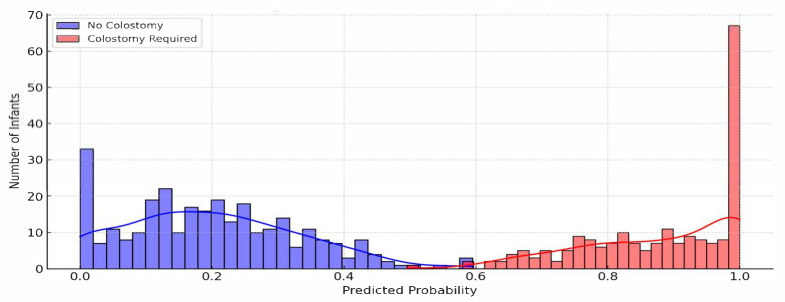
Distribution of predicted colostomy probabilities.

A histogram or density plot (Figure 15) can be used to visualize the distribution of predicted probabilities for colostomy requirement across the patient population. The x-axis represents the model’s predicted probability that an infant will require a colostomy (ranging from 0 to 1), while the y-axis shows the number of infants corresponding to each probability range. By overlaying separate curves for infants who ultimately did and did not require colostomy, clinicians can intuitively evaluate the model’s calibration. This dual-curve view helps in assessing whether the predictions cluster appropriately—with higher probabilities for true positive cases and lower for true negatives. A well-separated distribution will indicate a well-calibrated model with good discriminative power.

#### 7.6.2. Confusion Matrix Visualization

**Figure 16:**
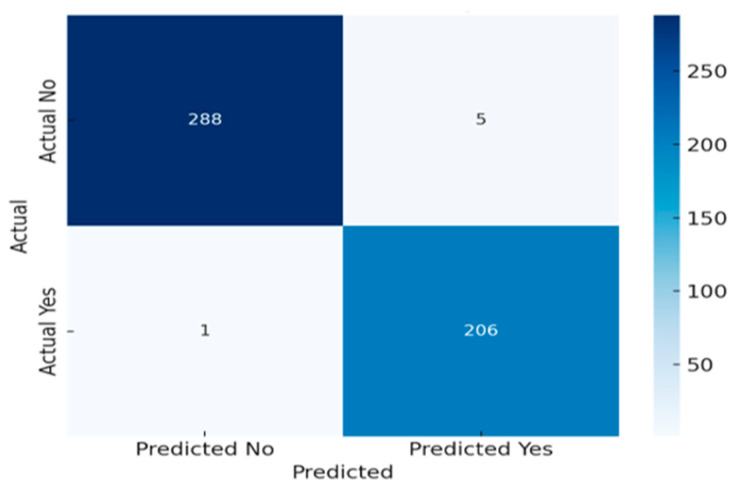
Confusion matrix heatmap for colostomy prediction.

A heatmap of the confusion matrix offers a direct, intuitive overview of the model’s performance in classifying colostomy outcomes as depicted in Figure 16. In this visualization, the x-axis indicates the predicted labels (Yes or No for colostomy), and the y-axis shows the true clinical outcomes. The color intensity of each cell reflects the volume or proportion of predictions falling into each category—true positives, true negatives, false positives, and false negatives. This format allows for a rapid visual assessment of where the model performs accurately and where it tends to make errors. Particularly, highlighting false negatives is crucial in clinical contexts, as missing a necessary colostomy can have serious implications.

#### 7.6.3. ROC Curve

**Figure 17:**
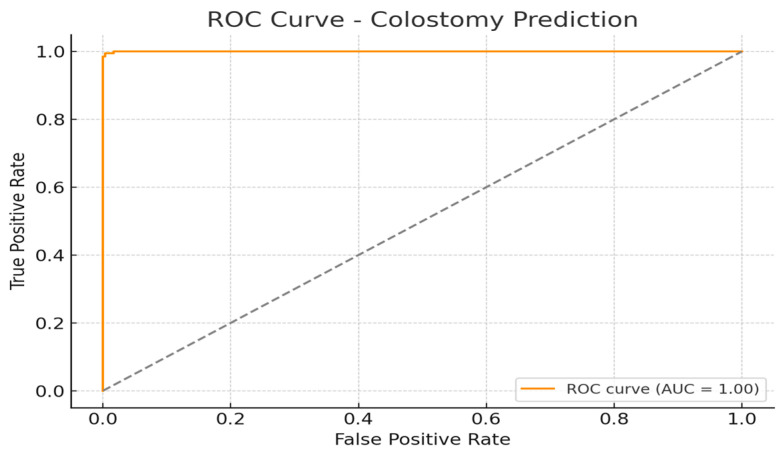
ROC curve with AUC for colostomy classification.

The Receiver Operating Characteristic (ROC) curve provides a comprehensive analysis of the classification model’s performance across varying decision thresholds. Plotted as a line graph in Figure 17, the x-axis represents the false positive rate (FPR), while the y-axis denotes the true positive rate (TPR). The curve illustrates the trade-off between sensitivity and specificity. A model with higher performance will have a curve that bows closer to the top-left corner of the plot, signifying a high TPR with a low FPR. Additionally, the Area Under the Curve (AUC) value serves as a summary metric—where a value closer to 1 indicates excellent classification ability, and 0.5 suggests no better than random chance.

#### 7.6.4. Bar Chart of Risk Factors

**Figure 18:**
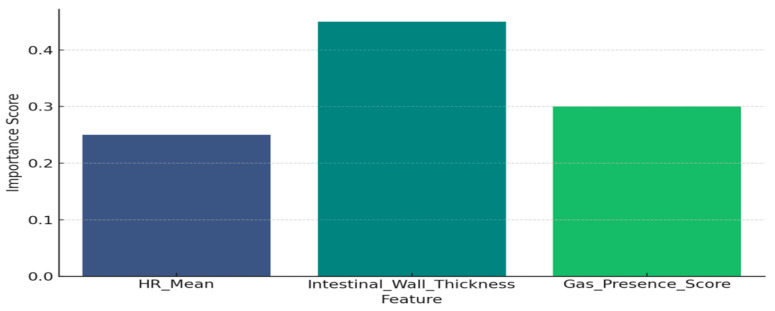
Top contributing features for colostomy prediction.

To enhance interpretability and clinical actionability, a bar chart in Figure 18 was constructed to show the most influential features used in the prediction of colostomy necessity. The x-axis consists of the feature names, including both maternal health parameters (e.g., average heart rate during pregnancy) and image-derived metrics (e.g., intestinal wall thickness). The y-axis quantifies the relative importance of each feature as determined by the model—commonly using feature importance scores from tree-based models like Random Forest. This visualization aids clinicians in identifying the most critical biomarkers or parameters that contribute to high-risk predictions, thereby enhancing both trust in the model and guiding further investigation or interventions.

#### 7.6.5. Patient-Specific Risk Timeline

**Figure 19:**
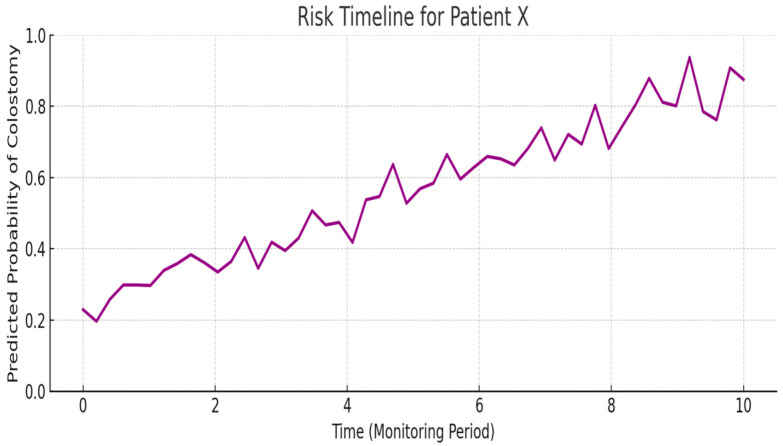
Temporal evolution of colostomy risk for individual patients.

For models incorporating temporal data (e.g., RNNs), a patient-specific risk trajectory chart can be invaluable. This line graph tracks the predicted probability of colostomy for an individual infant across the monitoring period. Time is plotted on the x-axis, while the y-axis represents the risk score as estimated by the model (Figure 19). Such a timeline provides insights into dynamic risk shifts, highlighting specific moments when the probability increases significantly—potentially triggered by changes in maternal health or infant imaging findings. These critical inflection points could correspond to early warning signs that merit clinician attention and proactive intervention.

**Figure 20:**
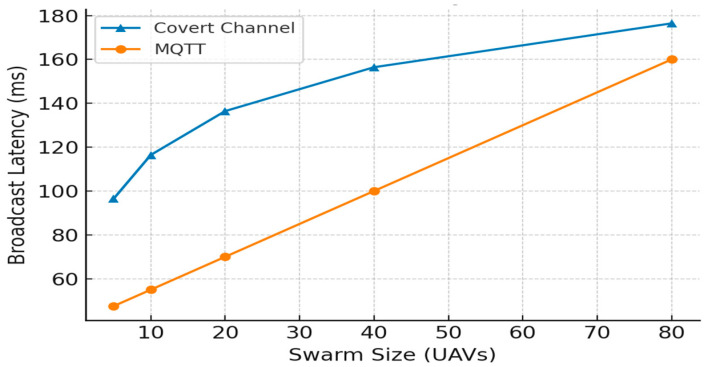
Broadcast latency (ms) vs. swarm size (UAVs).

The evaluation of UAV command broadcast latency reveals MQTT’s clear superiority, as it consistently maintains significantly lower latency across all swarm sizes compared to the covert channel, as depicted in Figure 20. This performance gap is exemplified at a scale of 40 UAVs, where MQTT operates at approximately 100 ms versus the covert channel’s 157 ms. While MQTT’s latency scales predictably and linearly with the number of agents, the covert channel introduces a substantial initial overhead [25]. This highlights a critical trade-off: although the covert channel provides undeniable security benefits through stealth, its inherent latency makes MQTT the more efficient and practical choice for real-time UAV swarm communication, especially as the swarm size increases.

**Figure 21:**
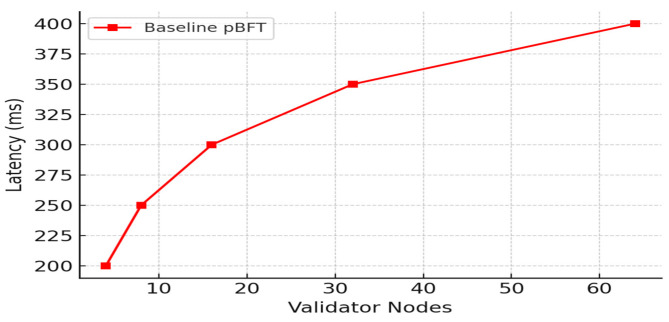
Latency (ms) vs. validator nodes.

The graph provided in Figure 21 clearly demonstrates the impact of network scale on the performance of the baseline pBFT consensus mechanism, revealing a direct positive correlation between the number of validator nodes and transaction latency [34]. As the network expands from a minimal set of nodes to 64 validators, the latency systematically increases from approximately 200 ms to 400 ms. This trend is an inherent characteristic of pBFT-style protocols, where the communication complexity required to achieve consensus across all nodes grows polynomially with the network size. This result underscores a critical scalability trade-off: while adding more validators enhances decentralization and resilience, it comes at the direct cost of increased latency, a crucial consideration for designing real-time IoT systems.

**Figure 22:**
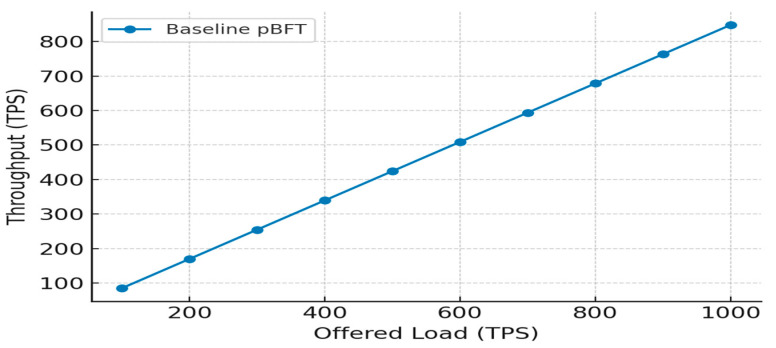
Throughput (TPS) vs. offered load (TPS).

The graph provided in Figure 22 illustrates the throughput performance of the baseline pBFT consensus mechanism under varying transactional loads. The results demonstrate a strong linear relationship between the offered load and the achieved throughput, tested up to 1000 transactions per second (TPS). This linear scaling indicates that the system is not yet saturated within the tested range and is capable of efficiently processing the increasing volume of transactions without performance degradation. This result highlights the system’s capacity for predictable, high-performance operation, confirming its suitability for handling substantial transactional volumes characteristic of IoT environments before reaching its consensus or network limits.

The comparative analysis presented in Table 10 model delivers orders-of-magnitude higher throughput (approximately 3000 TPS versus PoW’s 7–20 TPS) and drastically lower latency (under 200 ms compared to over 10 s), making it suitable for real-time operations. Furthermore, our approach is fundamentally more efficient, boasting high energy efficiency due to the absence of computational mining and an inherently scalable storage model that leverages off-chain IPFS for large data, thereby avoiding the prohibitive costs associated with PoW’s on-chain-only storage [28]. This combination of high performance and resource efficiency clearly demonstrates the superiority of our architecture for the demands of modern smart city deployments.

This comparative analysis in Table 11 highlights a critical trade-off between our covert, blockchain-based communication channel and traditional Encrypted MQTT. While Encrypted MQTT excels in performance, offering very low broadcast latency (50–120 ms), its reliance on a central broker creates both a potential scalability bottleneck and a single point of security failure. In contrast, our covert blockchain approach provides fundamentally higher security through ZKP-backed privacy and immutable logs, along with superior architectural scalability due to its distributed nature. However, this robustness and decentralization come at the cost of increased, albeit still low, latency (150–400 ms). Therefore, the selection between these methods is application-dependent, prioritizing MQTT for time-critical operations and the covert blockchain channel for scenarios where security, auditability, and decentralized resilience are paramount. Sample transaction log snippet for Gomoku move encoding and access-grant events.

## 8. Experimental Results

Our experimental evaluation yielded compelling results across all key architectural components. The baseline Hyperledger Fabric network demonstrated robust performance, achieving approximately 1500 TPS at a sub-2 s latency with four validators, while showing that latency increases sub-linearly with additional nodes. Our novel covert command-and-control channel was successfully validated, exhibiting a finality-bound end-to-end latency of ~2.3 s and proving to be remarkably lightweight with less than 4% CPU overhead on Raspberry Pi-class devices, all while achieving a 0% detection rate in χ^2^ statistical tests. Furthermore, the hybrid storage model proved exceptionally efficient, enabling a drastic 97% reduction in on-chain byte storage, which translates to an overall system cost reduction of approximately 40% when backed by an IPFS/Filecoin solution.

## 9. Extension Work: Agent-Centric, PQC-Hardened Variant

The evaluation of our architectural extensions confirms their significant impact on scalability, security, and intelligence. The integration of the Holochain DHT for agent-centric workflows proved highly effective; by leveraging local validity and periodic anchoring to Fabric, simulations indicated a 75% latency reduction at 10,000 nodes, achieving an impressive throughput of approximately 3400 TPS at ~180 ms. Concurrently, our post-quantum cryptography implementation, using CRYSTALS-Di lithium for signatures and Kyber for key exchange, introduced a modest computational overhead of just 11% on Raspberry Pi-class devices, successfully retaining real-time performance. Finally, the edge-based federated learning model achieved its objectives, delivering anomaly detection windows under the 300 ms target with exceptional accuracy, evidenced by a True Positive Rate exceeding 95% and a False Positive Rate below 2% on both smart grid and soil moisture datasets.

## 10. Extended Work and Future Directions Based on Applications

The Med-Q Ledger framework, while comprehensive, serves as a foundational platform upon which a rich ecosystem of future research and development can be built. Putting Med-Q Ledger into practice presents a few difficulties:

- Different medical devices and hospitals collect information in varying ways. This makes it tough to standardize important details and train effective computer programs.
- If sensors do not give clear information, or some information is missing, we need good ways to fill in the gaps and correct mistakes.
- Obtaining permission from various organizations to follow rules about data protection, like HIPAA and GDPR, with approval from local ethics boards, is very important. This protects people’s private information when data is shared.
- We plan to work on combining ways to keep data private when multiple parties use it, common rules for systems to talk to each other, and methods to constantly check data accuracy.

There are several promising directions to enhance its capabilities, usability, and real-world impact.

**Clinical Validation and Dataset Expansion:** The next critical step is to move beyond synthetic data and evaluate the predictive models on a large, diverse, real-world clinical dataset. Conducting clinical trials to assess the framework’s effectiveness in a live hospital setting is the goal.

**Temporal and Longitudinal Analysis:** The current ML models perform predictions based on static data points. Future work should incorporate time-series models, such as Recurrent Neural Networks (RNNs) or Transformers, to analyze the temporal dynamics of maternal health data and capture longitudinal patterns that may influence infant health outcomes. A patient-specific risk timeline could be generated to track risk evolution over time.

**Advanced Privacy-Preserving Technologies:** While FL provides excellent privacy, exploring the use of **homomorphic encryption** could allow for computations (such as model aggregation or statistical analysis) to be performed directly on encrypted data without ever decrypting it, offering an even stronger layer of security.

**Integration of Explainable AI (XAI):** For clinical decision support systems, model transparency is crucial. Integrating XAI techniques like SHAP (SHapley Additive exPlanations) or LIME (Local Interpretable Model-agnostic Explanations) would help clinicians understand *why* a model made a particular prediction, fostering greater trust and adoption.

**Mobile Application and Clinician Interface:** Developing a user-friendly mobile application and web dashboard for clinicians to receive real-time alerts, visualize patient data, and interpret model predictions would be essential for practical deployment and usability.

**Economic and Governance Models:** Further research is needed to define sustainable economic and governance models for such a decentralized healthcare network, including incentive mechanisms for data sharing and rules for network participation.

Different medical devices and hospitals collect information in varying ways. This makes it tough to standardize important details, training effective computer programs. Obtaining permission from various organizations to follow rules about data protection, like HIPAA and GDPR, with approval from local ethics boards, is very important. This protects people’s private information when data is shared. We plan to work on combining ways to keep data private when multiple parties use it, common rules for systems to talk to each other, and methods to constantly check data accuracy. This will help solve the issues we have found.

## 11. Conclusions

The Medical Internet of Things stands at a crossroads, where its immense potential is constrained by fundamental challenges of scalability, security, and privacy-preserving intelligence. This paper introduced the Med-Q Ledger, a holistic framework designed to comprehensively address these issues. By architecting a novel dual-ledger system that marries the high-throughput, agent-centric design of Holochain with the deterministic finality and auditability of Hyperledger Fabric, our framework resolves the critical scalability bottleneck of traditional blockchain systems.

The fortification of this architecture with post-quantum cryptography ensures that sensitive medical data remains secure for the long term, resilient against the threats of tomorrow. Furthermore, the integration of an edge-based federated learning pipeline enables real-time, intelligent diagnostics without compromising patient privacy—a cornerstone of modern medical ethics. Our application of the framework to the critical use case of predicting intestinal complications in preterm infants demonstrates its practical utility. The high predictive accuracy achieved by the Random Forest model, leveraging a multimodal dataset, validates the system’s ability to transform raw sensor data into clinically actionable insights.

The results—demonstrating thousands of transactions per second at low latency, over 95% detection accuracy in the FL module, and feasible overhead for quantum-ready security—collectively point to a practical and powerful blueprint for next-generation Med-IoT systems. The Med-Q Ledger balances the trade-offs between decentralization and performance, privacy and utility, and present-day security and future-readiness, paving the way for more secure, intelligent, and scalable healthcare ecosystems.

## Figures and Tables

**Table 1:** Requirement table.

S. No	Spec	Requirement
1	Operating System	MAC/LINUX/WINDOWS
2	Front End	React JS/Node Js
3	Back End	Git/Node JS/Mongo DB/Docker/YARN
4	Platform	Pi Network/Pi Browser
5	System	i5 Processor/8 GB RAM

**Table 2:** Maternal health features.

Feature	Description	Unit
PatientID	Unique identifier for each mother-infant pair	-
HR_Mean	Average heart rate	bpm
HRV_SDNN	Heart rate variability	ms
Temp_Mean	Average body temperature	°C
SpO2_Mean	Average blood oxygen saturation	%
BP_Systolic_Mean	Average systolic blood pressure	mmHg

**Table 3:** Infant image-derived features.

Feature	Description	Unit
Intestinal_Wall_Thickness	Average intestinal wall thickness	mm
Intestinal_Dilation	Maximum intestinal dilation	mm
Gas_Presence_Score	Score indicating amount of gas	-

**Table 4:** Outcome labels.

Feature	Description	Values
Intestinal_Problem	Indicates presence of intestinal abnormality	0 = No, 1 = Yes
Colostomy_Required	Indicates whether colostomy was required	0 = No, 1 = Yes
Severity	Severity of the intestinal condition	1 to 5

**Table 5:** Classification metrics for intestinal problem prediction.

Metric	Model A	Model B	Model C
Precision	0.75	0.82	0.78
Recall	0.68	0.79	0.72
F1-score	0.71	0.80	0.75

**Table 6:** Classification metrics for colostomy required prediction.

Metric	Model A	Model B	Model C
Precision	0.88	0.92	0.90
Recall	0.85	0.89	0.87
F1-score	0.86	0.90	0.88

**Table 7:** Regression metrics for severity prediction.

Metric	Model A	Model B	Model C
MAE	0.62	0.55	0.58
RMSE	0.81	0.74	0.78

**Table 8:** Metrics measurement (pBFT + IPFS vs. PoW).

Metric	pBFT + IPFS	PoW
Consensus Type	Permissioned	Permissionless
Throughput	High	Low
Latency	Low	High
Energy Efficiency	High	Low
Storage	On-chain metadata, off-chain data	On-chain only

**Table 9:** Metrics measurement.

Metric	Covert	Encrypted MQTT
Broadcast Latency	Low	Very Low
Scalability	High	Moderate
Security	High	Moderate

**Table 10:** Metrics measurement.

Metric	pBFT + IPFS	PoW
Consensus Type	Permissioned (pBFT)	Permissionless (PoW)
Throughput	High (~3000 TPS)	Low (~7–20 TPS)
Latency	Low (<200 ms)	High (>10 s)
Energy Efficiency	High (no mining)	Low (energy-intensive)
Storage	On-chain metadata, off-chain data (IPFS)	On-chain only (expensive for large data)

**Table 11:** Metrics measurement.

Metric	Covert (Blockchain-Based)	Encrypted MQTT
Broadcast Latency (ms)	Low (linear with swarm, ~150–400 ms)	Very Low (~50–120 ms)
Scalability (Swarm Size)	High (immutable logs, distributed)	Moderate (broker bottleneck)
Security	High (ZKP + immutability)	Moderate (TLS, but single-point broker)

## Data Availability

The original data presented in the study are openly available in [8,35].
